# The Porcelain Art in Dentistry

**Published:** 1903-11

**Authors:** Charles C. Patten

**Affiliations:** Boston, Mass.


### International Dental Journal.

Vol. XXIV. November, 1903. No. 11.

### Original Communications.[1]

### THE PORCELAIN ART IN DENTISTRY.[2]

BY CHARLES C. PATTEN, D.D.S., BOSTON, MASS.

The introduction of porcelain in its present form as a filling-material has been accompanied with more than ordinary interest and enthusiasm. In fact, it has been the all-absorbing subject of demonstration and discussion, both in public and private, for the past few years, and still continues to be one of the important features at all conventions. It has been considered by some who are as yet untouched by the fever as a mere fad that in the process of time would pass into oblivion. It is safe to say that the experimental stage has long since passed, and in the hands of careful, artistic operators porcelain is being made use of in such a way that nothing of which we at present have any knowledge can supplant it. That it has its points of weakness and defects its advocates do not attempt to deny. But who can name any one material with which we are familiar that is perfect in an all-

[1] The editor and publishers are not responsible for the views of authors of papers published in this department, nor for any claim to novelty, or otherwise, that may be made by them. No papers will be received for this department that have appeared in any other journal published in the country.

[2] Read before the Massachusetts Dental Society, Boston, Mass., June 3 and 4, 1903.

round capacity? Human nature in our profession is not unlike that found in the other professions, or, in fact, in any walk in life. It is one of the twentieth century characteristics that we like to ride on the top wave of enthusiasm, and this is especially true if it be accompanied by an equally high wave of prosperity. If pursuing a fad brings prosperity, and incidentally popularity and wider fame, who can be censured for cultivating it? But, gentlemen, porcelain art in dentistry is no fad. It is the very highest development of art in an effort to conceal the artificial in supplementing nature. The score of years just past, and, in fact, the last decade, have brought about marvellous developments in dentistry as well as the allied profession of medicine. It is fitting that one of the crowning achievements should be the introduction and practical application of a material for stopping cavities of decay in the natural teeth that more closely approximates nature than anything previously used for that purpose.

Predictions are frequently made in regard to the changes that time will show in the human race. Among others is the disappearance of the third molar, or wisdom-tooth, as it is generally known. Extensive examination of large numbers of Egyptian skulls show that the third molar is as large and well developed now as in the ancient races. It would be folly to prophesy that the time will ever come when the human teeth will not need scientific treatment to repair the ravages of decay. In view of this fact we are called upon to do this work of reparation in the most artistic possible manner. A dentist may develop a high order of skill as a manipulator of gold and incidentally save many teeth, yet be devoid of artistic sense in the slightest degree. He is a wonderfully developed mechanician, but not an artist. The dentist who meets with more than the ordinary success in prosthetic dentistry or in orthodontia must be an artist, or his work will not be artistic. We are confronted on every hand with the inartistic in dentistry.

The lifetime devotion of many of the pioneers in our profession to lift it to the highest level has not yet been crowned with entire success. It may be argued that only a small part of the public appreciate artistic dentistry, and sufficient compensation will not be received. To a certain extent this may be true, but the remedy lies in our own hands, and consists in constant efforts to educate our patients to a better understanding of what good dentistry is. That some people know and appreciate better work is evidenced by the interest shown in porcelain. To this class of patients the necessity for a display of gold is deplorable, and by many will not be tolerated. The growing interest shown by the public in porcelain work is made more manifest in the profession, and all are anxious to come into possession of an outfit and obtain the necessary knowledge to make use of it.

In view of what has been done from an educational standpoint in porcelain within the past three years, it seems inexcusable that any dentist within the pale of civilization should be ignorant on the subject. Everything that can be has been done by clinicians, demonstrators, essayists, and manufacturers to place it within the reach of every practitioner. The different kinds of enamels, bodies, furnaces, and tools to work with is almost bewildering. The display is so great as to be well-nigh discouraging at the outset for beginners. At any of our large conventions may be seen demonstrations of porcelain and furnaces of every form and make.

The advocates of high- and low-fusing enamels apparently meet with equal success. By what means can one determine which methods to adopt? There is only one positive way, and that is to give them all a trial. This would, of course, require not only a considerable expense, but much time. However, it is time and money well spent, as both high- and low-fusing bodies have their points of excellence, and there is much advantage in being able to make use of them as necessity arises. By far the most rapid work in porcelain can be done with Jenkins's enamel, using platinum as a matrix. It can be handled without investment, and the enamel will fuse quickly in the Hammond electric furnace with the lever on first step of rheostat and muffle wide open. If gold is used it must be invested in asbestos, and this requires more time and heat. In the high-fusing bodies the lever must be carried over to the fourth or fifth step for sufficient heat.

The success in using platinum as a matrix—and by this statement it is of course inferred that the whole operation must be successful—is governed by two things,—the platinum must be right and the operator able to manipulate it. These two conditions being favorable, the fit will be equally as good as though gold were used. In either case not only a high order of manipulative skill is necessary, but a thorough knowledge of cavity preparation. Without a properly shaped cavity no man on earth can make a successful porcelain filling. It is one of the details of the work that cannot be learned at one demonstration, or, in fact, at many. If done as it should be, something besides dull chisels and excavating burs must be used. In this, as well as all other operations where burs are used, they must be sharp and keen cutting, and a good outfit of finishing burs and Arkansas stones of all shapes and sizes kept on hand. All cases differ in some degree, and are governed by environment. One feature common to all and equally important with any other is that the enamel margins must be square and well defined and as smooth as it is possible to make them. In cavities of extreme depth, where it seems unnecessary to cut the enamel margins back to a level of the floor of the cavity after removing all signs of decay, a small quantity of cement may be introduced, which after setting can be shaped as though part of the tooth itself. When it becomes necessary to fill small but conspicuous cavities, do not hesitate to make them of sufficient size and depth to obtain good results. If an absolutely circular shape to the margins seems most desirable, be sure that one portion of the floor be made slightly deeper than the other, and no confusion will be experienced when the porcelain is ready to cement into place. Without some such precaution much time will be lost when time is precious in trying to decide which way the piece should enter the cavity. Labial cavities extending to and under the gum should be first packed with gutta-percha until the gum is well forced back, exposing the margins for proper treatment.

Do not attempt approximal cavities in bicuspids and molars involving the grinding surface unless the latter portion can be made strong and thick, with no thin edges to be chipped in mastication, and be sure that the occlusion is made correct. In approximal cavities of whatever class be sure to have plenty of space secured by previous wedging. Cavities of this class that under some conditions seem to be and are complicated become of the simplest character after proper separation. The statement was made in the writer's presence by a gentleman travelling about the country as an expert worker in porcelain, that little if any separation is needed in filling approximal cavities; in fact, much less than for gold. Such an assertion bears the stamp of ignorance and inexperience, and can be disproved by any practical demonstration. In labial cavities the gum takes more kindly to porcelain than any material that can be used. The slight gingival irritation is seldom present; secretions do not collect or remain on it. Frail teeth needing the support of cement are most happily restored to a condition of harmony and usefulness. Irritation from thermal changes is reduced to a minimum, or, in fact, entirely eliminated. For teeth below medium in structure, and there are many of them that we are called upon to treat and repair, where the best work we can do will be but temporary with any of the other materials, porcelain will do years of service. Its value in repairing the teeth of young patients is beyond question.

There is much to be learned about fusing or baking porcelain bodies and enamels. While it is possible to give a few words of advice or make a few suggestions in a short paper, experience is the only true way to learn how to obtain the best results. Conditions of absolute neatness and cleanliness must attend the manipulation and use of all porcelain powders. Anything in the way of carelessness or sloppiness will surely result in failure. Not only the hands, but the mixing-slabs, tools, and brushes, must be scrupulously clean and free from particles of dust, wax, or metal scraps of any nature. Better results are sometimes secured by more thoroughly triturating the bodies in a wedgewood mortar. This is especially true if the admixture of different kinds of bodies is required.

Furnaces are made to suit almost every requirement. Where electricity is available the electric furnace is undoubtedly the best. It is compact in form, clean, noiseless, and free from odor. After a thorough acquaintance it does the work in as perfect a manner as possible. While the first cost may be somewhat greater than others, the expense for running it is slight. With reasonable care and common sense the bills for repairs need not be large. The amount of heat is so easily controlled that all porcelain powders, from the lowest to the highest, may be accurately fused. There are also sold properly constructed muffles for use with the gas blowpipe and gasolene. The gasolene furnaces generate a high degree of heat, and are a boon for practitioners where gas and electricity are not available.

In order to obtain good results with any furnace the porcelain powders must be packed as firmly as possible and well dried out before being exposed directly to the heat. If this precaution be not observed, porosity will surely result. A slow bake is at all times better than a rapid one. In using the electric furnace extreme watchfulness is always required that the porcelain be not over-fused and the colors dissipated. This is in many cases the reason why the porcelain does not come from the muffle true to the selected shade.

It is an old adage that comparisons are odious, but even if so they are sometimes made with good results. That a thing or an idea is new and novel is not always a good and sufficient reason for adopting it. To be good and useful as well as new it must possess substantial advantages. That porcelain has in many respects advantages over other filling-materials there can be no question, and we are all certainly desirous of knowing exactly what they may be and how we can advance our usefulness by making use of them.

An eminent gentleman of our profession recently read a paper before the Ontario Dental Society in Toronto, the subject of which was "A Comparison of the Relative Merits of Gold Fillings and Porcelain Inlays." He started out by stating three important functions a filling must possess to be a success. The first is to check the ravages of caries. Any one of the materials with which we are familiar will do this with more or less success according to surrounding conditions and as they are indicated. No one would be justified in expecting equally successful results in using cement where gold was indicated. On the other hand, in many cases where gold has proved a failure cement has been a success in checking the ravages of caries. In these particular cases, always assuming that they are so situated that porcelain is available, it is one of the very best fillings to use.

The second function is to restore lost tissue. Of course, this means in as permanent a manner as possible. The second demand is fully covered by the first, in which he asserts that caries must be checked by a filling. That a filling ceases to restore lost tissue when it shows signs of recurrent decay is a self-evident fact. In many cases this recurrent decay will appear within a short time, in others perhaps never. At all events, we are bound to find it more or less, and much of our time is occupied in combating it, and generally by means of renewing the filling from a new foundation or adding to it where tooth-tissue is lost. In such cases there is reasonable room for doubt as to which filling shall be considered the more successful,—the one that not only checks the ravages of caries and restores lost tissue, but also restores the harmonious and natural appearance of the tooth as no other material can, and is then displaced, leaving very little if any signs of recurrent decay, the cavity being well protected by its uniform lining of cement, or the gold filling that resists displacement for perhaps no greater period of time, and may be found to be thoroughly undermined by recurrent decay.

In the third and last function, that harmony and natural expression be restored, the essayist admits that porcelain has preeminently the advantage. This from his point of view is the only one which justifies its use. This one alone, if there were no others, is a strong one to recommend it. But there are others that were not considered by him. No claim is put forth that a large corner of porcelain on one of the anterior teeth, where there is an edge to edge occlusion, or a large grinding surface on the posterior teeth, will stand the same amount of stress and hard usage that heavy gold carefully worked will. But we are justified in claiming, and experience and observation substantiate the claim, that a majority of the conspicuous contour fillings of gold in the anterior teeth can be and are being replaced by men skilled in the art of using porcelain, not only to the great and lasting satisfaction of patients, but to a degree of permanency that compares favorably with gold work in the same place.

[1] Read before The New York Institute of Stomatology, May 5, 1903.

